# Is quality costly? Patient and hospital cost drivers in vascular surgery

**DOI:** 10.1186/2191-1991-3-22

**Published:** 2013-10-21

**Authors:** Marie Kruse, Jan Christensen

**Affiliations:** 1 KORA: Danish Institute for Local and Regional Government Research, Købmagergade 22 DK-1150 Copenhagen, Denmark; 2 Local Government Denmark, Weidekampsgade 10 DK-2300 Copenhagen, Denmark

**Keywords:** I12- health production, C33 - Models with Panel Data, Longitudinal Data, Spatial Time Series, D24 - Production, Cost, Capital, Capital, Total Factor, and Multifactor Productivity, Capacity, Hospital costs, Treatment quality, Cost drivers, Vascular surgery

## Abstract

An increasing focus on hospital productivity has rendered a need for more thorough knowledge of cost drivers in hospitals, including a need for quantification of the impact of age, case-mix and other characteristics of patients, as well as establishment of the cost-quality relationship.

The aim of this study is to identify cost drivers for vascular surgery in Danish hospitals with a specific view to quality of the treatment: Is higher quality associated with increased costs, when all other cost drivers are accounted for?

We analyse cost drivers in a register-based study, using patient level data from three sources: The Vascular Register, the hospital cost database, and the National Patient Register with added DRG-information. The analysis follows a multilevel set-up, where cost drivers at patient level are analysed in a set of general linear regression models including complications and mortality as quality measures. At the hospital level of the analysis, we analyse deviations of observed costs from risk-adjusted costs and compare these to deviations of observed quality from risk-adjusted quality.

We find, not surprisingly, that a number of patient characteristics, including case-mix and severity, have a major impact on treatment costs. At patient level, both complications and mortality are associated with increased costs. At hospital department level, results are not straightforward, but could indicate a U-shaped association.

We conclude that the relation between costs and quality is not straightforward, at least not at department level. Our results indicate, albeit vaguely, a U-shaped relation between quality, in terms of fewer surgical complications than expected, and costs at department level, since our results suggest that increasing costs for vascular departments are associated with increased quality when costs are high and decreased quality when costs are low. For mortality however, we have not been able to establish a clear relation to costs.

## Background

Scarcity of health care resources and attempts at increasing competition between hospitals have put an increased focus on hospital productivity. In Denmark, analyses of hospital productivity date back almost 20 years [1], at that time driven by a pure academic interest. In more recent years, policy measures have aimed at creating incentives for increases in productivity [2,3]. Broadly speaking, inter-hospital differences in productivity can be caused by either different values of production (output differences), or cost (input) differences. This study focuses on the latter.

We analyse cost differences between hospitals by identification of cost drivers at two levels: patient level and department level. A similar study of English obstetrics treatment [4,5] found that patient level characteristics constitute the most important cost drivers. In this study we aim to take the analysis a step further and introduce treatment quality in the model. Previous research has shown that quality – measured as the absence of complications – may be among the most important cost drivers for hospitals [6-8]. These analyses define quality by various measures of negative outcomes available in existing registers. Gutacker et. al. [9] include additionally a more direct health effect, based on self-reported pre- and post-measurements of the health status. This type of information is not available for the present analysis, which relies solely on register-based data.

The actual relation between costs and quality is not established, however. Neither has any kind of optimal level of quality that outweighs the costs and benefits of producing particular levels of quality of treatment been determined. Improving quality may be costly, but poor quality may also render higher costs, as suggested by e.g. [10]. Analysing the relation at different levels has proven to entail results apparently at odds with each other. Based on the same data source, [11] established a negative correlation at patient level, whereas [12] found a positive correlation at hospital level. It is possible, that the quality-related costs in fact follow a U-shaped curve, as suggested by [6,9]. This essentially means that for low levels and high levels of quality, the associated costs are high, while at some intermediate level (at the minimum point of the U-shaped curve) of quality costs are minimized. If that is the case, the next challenge is to identify what part of the curve is reflected in our data: If observations from a department indicates a downward sloping relation between costs and quality, then an increase in quality would reduce costs, because the observed level of quality is below the optimal level. If, on the other hand, the relation between costs and quality is positive, then this is insufficient information to show quality is at a suboptimal level.

Results from the EuroDRG group have established that quality aspects have an impact on treatment costs [13]. For 10 diagnosis-related groups of patients cost driving factors are identified. Significant effects of adverse events are found. For most diagnosis groups, wound infections are associated with increased patient treatment costs [14], pointing towards a negative cost-quality association.

Whereas all the analyses mentioned above estimate relations between costs and quality using the cost side as the dependent variable, as is done in the present analysis, other studies [15,16] model the relation the other way around.

Danish hospital data allow for detailed analyses at individual level. In Denmark, all individuals have a social security number which isused throughout public registers and databases. This allows us to analyse cost data and output information at individual level, while taking clinical and personal information into account. Hence, the data provide a good basis for analysis of cost drivers at the individual level. Due to the small size of the country, and consequently the low number of hospitals, analysis of Danish data at hospital level constitutes a challenge. Another challenge for analysing hospital level cost drivers is the absence of a common production function across hospitals [4], related to hospitals differing in size and scope.

In this study, we concentrate on vascular surgery in order to overcome some of the size and scope differences between hospitals. Vascular departments are chosen due to the availability of detailed data on quality parameters. Hence, we compare departments and not hospitals. In addition, the analysed patient group appears more homogenous when only one medical specialty is analysed.

The disadvantage of limiting the analysis to vascular departments relates to sample size. We have sufficient data to analyse cost drivers at patient level. However we have data for only 11 hospital departments, rendering a number of challenges for the analysis of department level cost drivers. We tried to overcome these challenges by instead reporting department level results graphically.

The aim of this study is to identify cost drivers for vascular surgery in Danish hospitals with a specific view to quality of the treatment: Does quality increase costs, when all other cost drivers are accounted for?

### Data

**Figure 1:**
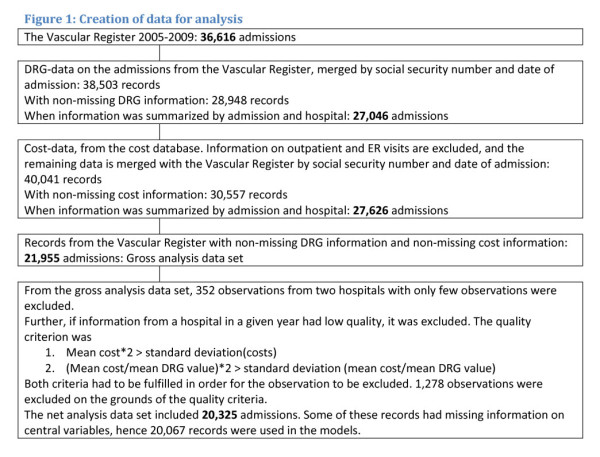
**Creation of data for analysis**.

For this analysis, we used a clinical database, the Vascular Register [17], comprising patients admitted for vascular surgery during 2005–09 at Danish hospitals. A wide range of variables are included, including information not available in usual patient registers, in particular ASA score for assessing the severity of illness, information on smoking habits, Body Mass Index (BMI) and registrations of adverse events, such as infections and death. The register contains about 55,500 discharges for about 38,500 patients. Data covers all visits. Our analyses focus on admission of inpatients, of which there are more than 36,000 discharges and hence both outpatient visits and emergency visits are excluded from the analysis of costs and quality. We linked information from the Vascular Register to information about costs, derived from the National Board of Health cost database. This source of data provides information on gender and age, as well as discharge specific costs, enabling targeted modelling of cost drivers on a patient level. The process of linking and filtering data for analysis is illustrated in Figure 1 below. Finally we linked this combined information to the DRG-tariff and DRG-weights for each discharge [18].

The Danish DRG-system contains close to 650 Diagnosis related Groups for inpatients, where only a minor subset is relevant for vascular surgery. DRG is used in this analysis as expression of case-mix. The groups are constructed such that they are clinically meaningful (based on diagnosis and treatment) and homogenous in terms of consumption of resources [1,18].

Information from the three sources was linked by the social security number and the start date of the treatment history. We allowed the start date to vary up to two days in order to capture inpatient visits initiated by either outpatient or emergency room (ER) visits. Since multiple admissions may be related to quality, we performed a sensitivity analysis excluding readmissions within 30 days.

The data set used for analysis was defined as: admissions during 2005–09 in the Vascular Register, with non-missing cost information from the cost database and non-missing DRG-information. For most departments data coverage was virtually complete for all years. Unfortunately, for some departments data coverage was only good for some years. In addition, one department had only observations for a single year and another department had very few observations, all data for these departments was excluded from the analysis.

Because of the variations in data coverage, we included only data for combinations of departments and years, if the coverage was good. In order to examine the quality of data coverage, we defined some inclusion rules at the department level. The first inclusion rule was that the standard deviation for costs should be less than twice the mean costs for each department in a given year. The second inclusion rule defined the same criterion for the mean cost divided by the mean DRG value. Hence, we excluded a number of years of observations for a group of departments, namely those that did not comply with both rules. These departments did however have valid observations in other years and were only excluded in those years where they did not adhere to the inclusion rules. Since the excluded data represent departments or years with very few data, the inclusion rules did not change much.

The analysis thus uses information on 20,325 admissions, cf. Figure 1. For 258 observations, information on central analysis variables were missing and these observations were excluded from the analysis. In a sensitivity analysis, we included observations from the two omitted departments and observations that didn’t comply with the inclusion rules. In the sensitivity analysis, 21,954 observations were included.

The cost information from the National Cost Database entails a great level of detail, because all major cost-driving events during an admission are recorded for each discharge. Costs at the discharge level constitute a sum of patient level costs and overhead costs distributed amongst the patients. Furthermore, the National Cost Database covers all discharges. Since all data is collected administratively, and permission to use for research purposes has been granted, no patient consent is needed.

## Methods

We analysed cost drivers in vascular surgery at patient level using a fixed effects generalised linear regression model. We chose to estimate the model without an intercept term, in order to establish department effects for all departments. All other variables still need reference terms because the department dummy variables act as intercepts for the reference patient.

Identification of cost drivers

(1) $$C_{\mathrm{ij}}=\sum {}_{x}\beta_{x}X_{\mathrm{ij}}+\sum {}_{y1}\beta_{y1}Y_{\mathrm{ij}}^{1}+\sum {}_{j}\beta_{j}Z_{j}+e_{\mathrm{ij}}$$

(2) $$C_{\mathrm{ij}}=\sum {}_{x}\beta_{x}X_{\mathrm{ij}}+\sum {}_{y2}\beta_{y2}Y_{\mathrm{ij}}^{2}+\sum {}_{j}\beta_{j}Z_{j}+e_{\mathrm{ij}}$$

Where subscripts indicate the following: patients i, departments j, patient level covariates x, y_1_ and y_2_, and time invariant department dummy variables z, *β*_*x*_, *β*_*y*1_, *β*_*y2*_ and *β*_*j*_ are vectors of parameters. *C*_i*j*_ are costs at patient level. *X*_i*j*_ is a vector of variables indicating the following patient characteristics: age (a set of dummy variables per 10 year age interval – reference category 60–70 years), gender (woman), smoking status: a dummy variable for daily smoking, 0 otherwise; Body Mass Index (dummy variables indicating the following BMI levels: less than 18 or underweight, 18–24 or normal weight (reference), 25–29 or overweight and more than 30 or obese), case-mix (reflected by the DRG-value of the individual discharge), dummies for severity reflected by the ASA score(American Society of Anesthesiologists physical classification system): missing, 1 (mild systemic disease and reference), 2 – severe systemic disease, 3 – severe and life-threatening systemic disease; and 4 – very moribound person not expected to survive without operation; a set of dummy variables for whether the patient is admitted acute or not (very acute, acute, subacute (reference), and elective) and finally a time effect, expressed by a variable indicating the year of treatment (2005 was reference).

The two *Y*_i*j*_ vectors represent quality, $Y_{\mathrm{ij}}^{1}$ being complications at patient level (surgical wound complications, other surgery complications, wound infections (e.g. haemorrhage), and general complications including heart or kidney problems, stroke or ICU admission), while $Y_{\mathrm{ij}}^{2}$ is 30 days mortality. We chose to analyse complications and mortality in two different models since mortality was highly correlated to complications. *Z*_*j*_ is a vector of department dummy variables.

The regression model described in formula 1 and 2 renders information on cost drivers. We expect to find that older patients may be more costly than younger [19], similarly for overweight and obese versus normal weight [20-22], and that smokers are more costly than non-smokers [23,24]. The parameters *β*_*y*1_ and *β*_*y*2_ indicate the impact of quality on patient level costs. The sign and magnitude of these parameters indicate the association between costs and quality at patient level.

We assessed hospital level productivity in a manner inspired by the approach taken by [4]. In their model of obstetrics treatment in the UK, Laudicella *et al*. illustrate department level variation in costs by plotting actual costs against risk-adjusted costs, or level of inefficiency [4]. When applying this approach in our study, we regard the risk-adjusted costs as equal to the parameter estimates *β*_*j*_ of the dummy variable *Z*_*j*_ in a regression model that accounts for all risk factors but exclude quality, hence:

(3) $$C_{\mathrm{ij}}=\sum {}_{x}\beta_{x}X_{\mathrm{ij}}+\sum {}_{j}\beta_{\mathrm{cj}}Z_{j}+e_{\mathrm{ij}}$$

The *β*_*cj*_ estimates can be interpreted as the department specific contribution to the cost level, since it explains the risk-adjusted costs, having taken all of the above mentioned variables, including patient case-mix but excluding quality, into account [6]. This type of unexplained deviation from expected costs is also referred to as the department level of inefficiency [9,16,25,26]. The department fixed effects *β*_*cj*_ are interpreted as risk-adjusted costs and used in the department level analysis.

At department level, we subtract the *β*_*j*_’s from the *C*_*j*_’s, in order to obtain an estimate of unexplained costs, or inefficiency. Since all patient level characteristics are included in the estimation, the resulting estimates could be interpreted as being risk-adjusted costs [7]. Hence, for the cost variable in the department analysis, we look at what could be called *additional* costs, that is, the difference between observed costs and risk-adjusted costs. If this figure is positive, there are costs that cannot be explained by patient risk factors or case-mix.

In a similar manner, we estimate risk-adjusted (or *additional*) complications – or quality - in a logit model specified by

(4) $$Q_{\mathrm{ij}}=\sum {}_{x}\beta_{x}X_{\mathrm{ij}}+\sum {}_{j}\beta_{\mathrm{qj}}Z_{j}+\vartheta_{\mathrm{ij}}$$

Here, the *X*_i*j*_ vector includes patient level characteristics, such as age, gender, etc. (as above), the *Z*_*j*_ vector is department dummies, and the *β*_q*j*_ are estimates of risk-adjusted quality. We used surgical complications and mortality as measures of quality, and multiplied these by −1, in order to obtain a measure that was high for high quality and *vice versa*. The logit model renders estimated probabilities of complications or death, and these are used for risk-adjusted complications or death below.

The department level analysis is based on a graphical approach, as in [6]. Here, we plot the difference between observed and risk-adjusted costs *C*_*j*_-*β*_*cj*_ against the difference between risk-adjusted complications and observed complications *Q*_*ij*_-*β*_*qj*_. Thus, a cost level higher than expected is interpreted as higher costs, while fewer complications than expected are interpreted as higher quality. Consequently, an observation in the North-Eastern quadrant expresses high costs and high quality.

If a department is primarily located in the North-Eastern or the South-Western quadrant, it can be interpreted as a positive association between quality and costs. If a department is primarily located in the South-Eastern or North-Western quadrant, the cost-quality association is negative [6].

Generally, when we analyse the impact of quality on costs at patient level, we would expect to find that high levels of complications are positively related to costs, since patients with complications are costly. The association between costs and mortality could go both ways. A high mortality could be inversely related to costs for two reasons. Firstly, patients dying at the hospital could have less time there and therefore being exposed to cost driving activities for a shorter time span. Secondly, resource prioritization could lead to patients dying. On the other hand, high mortality could be positively correlated with costs. Hospitals may spend more on patients at risk of dying, while employing additional effort attempting to save them, by using more complex or costly equipment and treatments.

Costs are reported as Euros, 2009-price level. Euros were computed from Danish Kroner using the exchange rate EUR 1 = 7.45 DKK, which is an average of annual exchange rates 2005–2009 derived from [27]. SAS™ v. 9.3 was used for all analyses.

## Results

In Table 1, data is described at department level. Cells with missing observations indicate that data from the particular department has been excluded in that year, due to poor quality of the data.

**Table 1:** Descriptive statistics

	**Department 1**	**Department 2**	**Department 3**	**Department 4**	**Department 5**	**Department 6**	**Department 7**	**Department 8**	**Department 9**	**Department 10**	**Department 11**	**Total population**
Number of discharges												
2005	271	718	135	660	514	790	815	433	644	139	6	5,125
2006	253	662	124	740	557	758	154	35	0	3	11	3,297
2007	232	742	133	822	557	790	836	428	2	0	83	4,625
2008	281	771	133	786	573	203	193	475	477	0	64	3,956
2009	366	799	133	942	681	796	153	504	531	0	46	4,951
Total	1,403	3,692	658	3,950	2,882	3,337	2,151	1,875	1,654	142	210	21,954
University hospital	no	yes	no	no	no	yes	yes	yes	no	no	no	
DRG index	0.90	1.05	0.78	0.97	0.91	1.13	0.99	1.16	0.92	0.86	0.74	1.00
Average cost per discharge, actual (DKK)	84,956	64,937	57,755	69,828	58,525	80,011	94,845	91,434	63,396	115,497	41,186	73,508
Average cost per discharge, predicted (DKK)	69,050	80,443	59,746	74,609	69,748	85,668	73,956	89,858	69,722	54,223	60,139	76,432
Number of vascular operations as a percentage of total at the department level	98.8	81.6	97.4	95.1	96.1	95.6	97.8	93.4	98.7	83.1	89.5	93.6
Average length of stay (days)	6.1	3.7	3.7	4.8	4.9	6.3	5.2	5.5	4.3	4.1	1.7	4.92
**Patients**												
Average age	66.0	62.7	68.3	68.2	67.5	66.2	66.5	67.2	67.4	65.7	67.3	66.4
Percentage male	59.4	60.8	57.9	57.0	58.3	62.4	59.8	61.9	53.6	59.9	56.2	59.3
Percentage smokers	47.1	27.0	38.9	35.5	41.4	45.0	37.8	42.3	38.9	36.6	21.9	38.1
Percentage with BMI >25	30.3	21.5	49.4	30.1	39.2	37.4	40.8	37.3	39.1	36.6	9.0	33.3
Percentage with ASA score > 2	40.8	11.5	45.9	29.2	27.4	56.4	29.3	19.9	19.5	28.2	32.9	29.9
Percentage admitted acute	3.7	19.5	1.0	27.7	19.4	46.2	36.1	27.5	26.1	11.3	5.2	26.1
30-day mortality, per cent of discharges	0.93	2.79	1.96	3.31	2.73	5.28	4.66	3.18	2.36	4.93	0.5	3.36
Wound complications, per cent of discharges	13.78	6.51	8.84	8.60	8.41	14.86	5.05	13.75	11.32	7.04	5.47	9.68
Infections, per cent of discharges	2.04	1.19	1.31	2.05	2.28	3.99	1.05	4.27	2.74	0	0	2.30
Surgical complications, per cent of discharges	1.76	2.87	2.45	4.13	3.43	6.99	5.38	5.20	4.35	5.63	0	4.26
General complications, per cent of discharges	3.70	5.46	4.26	7.34	4.34	14.75	8.38	7.23	6.03	9.15	1.49	7.44

NOTE: Some observations are excluded from the analysis due to poor quality and/or a small number of observations. All observations from departments 10 and 11 are excluded in the analysis. Inclusion rules are explained in the data section. Table 1 includes all observations in data.

There are some variations between departments. Not surprisingly, there are differences in costs and case-mix, also mirrored in the variations in length of stay, severity and acute admissions. There are no great variations in age and gender, but some differences in other risk factors. The quality variables, except for mortality, show rather high variation between departments, generally university hospitals have the highest share of complications.

In Table 2, the results of the first patient level model are shown. Here, cost drivers are identified at patient level. The four complication variables indicate quality in this model.

**Table 2:** **Cost drivers at patient level** – **quality included as four complications variables**

**Variable name**	**Parameter estimate (2009 EURO’s)**	**Standard error**	**t value**	**Probability of estimate = 0**
**Department 1**	1,728*	599	2.88	0.0039
**Department 2**	1,192	638	1.87	0.0615
**Department 3**	356	531	0.67	0.5027
**Department 4**	−187	523	−0.36	0.7200
**Department 5**	−743	527	−1.41	0.1590
**Department 6**	2,259*	523	4.32	< .0001
**Department 7**	−421	862	−0.49	0.6254
**Department 8**	1,772*	538	3.29	0.0010
**Department 9**	581	541	1.07	0.2827
**2006**	475	255	1.87	0.0620
**2007**	1,819*	222	8.19	< .0001
**2008**	367	241	1.53	0.1273
**2009**	1,142*	225	5.07	< .0001
**DRG index**	4,563*	245	18.88	< .0001
**DRG index squared**	637*	51	12.47	< .0001
**Age less than 50**	53	266	0.20	0.8424
**Age 50**-**60**	64	226	0.28	0.7760
**Age 70**-**80**	111	180	0.62	0.5372
**Age 80**-**90**	178	234	0.76	0.4470
**Age 90**+	−1,146	646	−1.77	0.0760
**Woman**	−520*	147	−3.55	0.0004
**Daily smoker**	−51	93	−0.55	0.5853
**BMI information missing**	1,066*	447	2.39	0.0170
**Underweight**	−233	437	−0.53	0.5937
**Overweight**	−41	193	−0.21	0.8300
**Obese**	318	259	1.23	0.2201
**Very obese**	−1,505	910	−1.65	0.0983
**Diabetes**	−105	105	−1.01	0.3143
**Cerebral comorbidity**	−193*	82	−2.35	0.0186
**Hypertension**	−399*	123	−3.26	0.0011
**Cardiac comorbidity**	15	53	0.28	0.7795
**Pulmonary comorbidity**	494*	175	2.82	0.0048
**Very acute admission**	2,306*	547	4.21	< .0001
**Acute admission**	857*	321	2.67	0.0077
**Elective**	589	300	1.96	0.0500
**Severity information missing**	−322	403	−0.80	0.4249
**Moderate (ASA = 2)**	−481*	201	−2.40	0.0166
**Severe (ASA = 3)**	−234	237	−0.98	0.3249
**Very severe/fatal (ASA > 3)**	1,266*	454	2.79	0.0053
**Lenght of stay**	493*	10	47.21	< .0001
**Wound complications**	606*	245	2.45	0.0145
**Surgical complications**	4,935*	367	13.46	< .0001
**Infections**	715	470	1.52	0.1281
**General complications**	5,296*	297	17.80	< .0001
**Number of observations**	20,067			
**R**^**2**^	0.63			

NOTE: Parameter estimates that are statistically significant at 5 per cent level have the suffix *.

Table 3 shows the results of the patient level model with mortality instead of complications. Otherwise, as model [1] and [2] reflect, the model specifications are identical.

**Table 3:** **Cost drivers at patient level** – **quality included as 30 days mortality**

**Variable name**	**Parameter estimate (2009 EURO’s)**	**Standard error**	**t value**	**Probability of estimate = 0**
**Department 1**	1,374*	607	2.26	0.0236
**Department 2**	994	646	1.54	0.1240
**Department 3**	144	538	0.27	0.7891
**Department 4**	−530	530	−1.00	0.3171
**Department 5**	−691	534	−1.29	0.1959
**Department 6**	2,044*	530	3.86	0.0001
**Department 7**	−753	874	−0.86	0.3889
**Department 8**	1,494*	545	2.74	0.0061
**Department 9**	442	548	0.81	0.4199
**2006**	561*	258	2.18	0.0296
**2007**	1,827*	225	8.11	< .0001
**2008**	314	244	1.29	0.1987
**2009**	1,093*	228	4.79	< .0001
**DRG index**	4,832*	245	19.75	< .0001
**DRG index squared**	709*	52	13.72	< .0001
**Age less than 50**	−42	270	−0.16	0.8764
**Age 50**-**60**	9	229	0.04	0.9690
**Age 70**-**80**	166	183	0.91	0.3642
**Age 80**-**90**	179	238	0.75	0.4505
**Age 90**+	−1,443*	654	−2.21	0.0275
**Woman**	−571*	149	−3.84	0.0001
**Daily smoker**	−85	94	−0.90	0.3681
**BMI information missing**	1,221*	453	2.70	0.0070
**Underweight**	−338	443	−0.76	0.4457
**Overweight**	−2	195	−0.01	0.9935
**Obese**	340	263	1.29	0.1960
**Very obese**	−1,378	923	−1.49	0.1354
**Diabetes**	−168	106	−1.58	0.1132
**Cerebral comorbidity**	−146	83	−1.76	0.0787
**Hypertension**	−357*	124	−2.87	0.0041
**Cardiac comorbidity**	33	53	0.61	0.5390
**Pulmonary comorbidity**	504*	178	2.84	0.0045
**Very acute admission**	2,340*	555	4.22	< .0001
**Acute admission**	1,217*	326	3.74	0.0002
**Elective**	736*	304	2.42	0.0155
**Severity information missing**	−322	409	−0.79	0.4301
**Moderate (ASA = 2)**	−476*	204	−2.34	0.0195
**Severe (ASA = 3)**	−267	241	−1.11	0.2681
**Very severe/fatal (ASA > 3)**	1,552*	464	3.35	0.0008
**Lenght of stay**	549*	10	53.59	< .0001
**30 days mortality**	3,872*	423	9.15	< .0001
**Number of observations**	20,067			
**R**^**2**^	0.62			

NOTE: Parameter estimates that are statistically significant at 5 per cent level have the suffix *.

The explanatory power of the two cost driver models is reasonable, with R^2^’s above 0.6. Patient case-mix, gender, and acute admission are important cost drivers, with increasing case-mix and cases of acute admission increasing costs. Most patient co-morbidity parameters are statistically significant as well. In both models, the quality variables, except for infections, are statistically significant cost drivers. All are positively related to costs.

**Figure 2:**
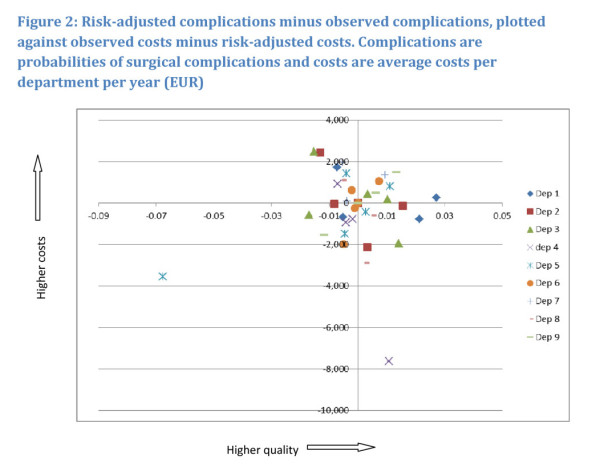
**Risk-adjusted complications minus observed complications, plotted against observed costs minus risk-adjusted costs**. Complications are probabilities of surgical complications and costs are average costs per department per year (EUR).

**Figure 3:**
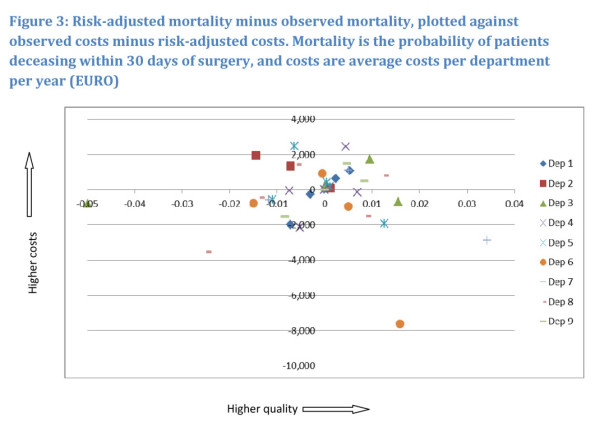
**Risk-adjusted mortality minus observed mortality, plotted against observed costs minus risk-adjusted costs**. Mortality is the probability of patients deceasing within 30 days of surgery, and costs are average costs per department per year (EURO).

In Figures 2 and 3, the observed quality (complications/mortality) minus the risk-adjusted quality is plotted against observed costs minus risk-adjusted costs. This is done for surgical complications as expression of quality (Figure 2) and mortality as expression of quality (Figure 3).

Hospitals located at the origin operate with costs and complications/mortality as expected, judged on the basis of their particular patient characteristics. In the North-Eastern quadrant hospitals have higher costs as well as high quality/low mortality, which seems to indicate that quality comes at a cost. In the North-Western quadrant hospitals have higher costs and lower quality/higher mortality than expected, indicating that low quality comes at a cost. In the South-Western as well as in South-Eastern quadrants hospitals have lower costs. In the former hospitals have lower quality/higher mortality and higher quality/lower mortality then average respectively. For most hospitals, it follows from the figures, results are ambiguous. All hospitals are located in at least 2 quadrants, with the majority of observations located around the origin. This seems to indicate that over the years hospitals adjust levels of cost and ways of operating that affect quality and mortality. It follows from Figure 2, that except for the observation with the lowest costs and the one with the lowest quality as outliers, all others are located within a U-shaped area, starting from the North-eastern part of the North-Eastern quadrant, including the corners of the South-Eastern and South-Western quadrants closest to the origin and ending with the North-Western part of the North-Western quadrant.

The U-shape in Figure 2 illustrates the association between additional costs, i.e. costs minus risk-adjusted costs, and additional quality, i.e. the share of surgical complications minus the risk-adjusted share of surgical complications. The cost figure being positive in Figure 2 indicates that costs are higher than what patient risk factors can explain, and similarly for surgical complications. This, in turn, seems to suggest that high costs for the vascular departments are associated with either increased quality or decreased quality, in other words, that high quality is costly and so is low quality. Within this U-shaped range, hospital 1 displays an almost uniformly positive association, with high costs associated with high quality. This does not, however, alter the overall picture. The relation between costs and mortality as depicted in Figure 3 has no such clear relation.

In Table 4 the patient level results of the two sensitivity analyses are shown. The first sensitivity analysis excludes all readmissions within 30 days (this leads to the exclusion of 883 observations), while the second used all observations from all departments. Hence, in this sensitivity analysis, no observations were excluded on the basis of data shortage or poor quality. Quality is represented by 30-days mortality in both sensitivity analyses.

**Table 4:** **Sensitivity analyses**, **patient level**

**Variable name**	**Parameter estimate (2009 EURO’s)**	**Standard error**	**t value**	**Probability of estimate = 0**
**Sensitivity analysis 1: Only the first discharge per patient per year is included**				
**Department 1**	696	618	1.13	0.2599
**Department 2**	668	658	1.02	0.3096
**Department 3**	−298	549	−0.54	0.5875
**Department 4**	−915	540	−1.70	0.0899
**Department 5**	−1,044	544	−1.92	0.0551
**Department 6**	1,588*	540	2.94	0.0033
**Department 7**	−1,152	905	−1.27	0.2034
**Department 8**	1,157*	555	2.08	0.0372
**Department 9**	49	558	0.09	0.9300
**2006**	480	263	1.82	0.0682
**2007**	1,888*	229	8.25	< .0001
**2008**	495*	249	1.99	0.0465
**2009**	1,206*	232	5.19	< .0001
**DRG index**	4,395*	249	17.65	< .0001
**DRG index squared**	744*	52	14.32	< .0001
**Age less than 50**	48	275	0.18	0.8610
**Age 50**-**60**	137	234	0.59	0.5585
**Age 70**-**80**	178	186	0.96	0.3375
**Age 80**-**90**	86	242	0.35	0.7227
**Age 90**+	−1,344*	668	−2.01	0.0444
**Woman**	−534*	151	−3.53	0.0004
**Daily smoker**	−100	96	−1.04	0.2983
**BMI information missing**	1,440*	459	3.14	0.0017
**Underweight**	−272	449	−0.61	0.5451
**Overweight**	37	198	0.19	0.8519
**Obese**	380	267	1.42	0.1542
**Very obese**	−1,505	946	−1.59	0.1116
**Diabetes**	−225	108	−2.08	0.0380
**Cerebral comorbidity**	−159	84	−1.89	0.0589
**Hypertension**	−380*	126	−3.01	0.0026
**Cardiac comorbidity**	20	54	0.37	0.7107
**Pulmonary comorbidity**	590*	181	3.26	0.0011
**Very acute admission**	2,748*	575	4.78	< .0001
**Acute admission**	1,247	334	3.74	0.0002
**Elective**	896	311	2.88	0.0040
**Severity information missing**	−126	420	−0.30	0.7640
**Moderate (ASA = 2)**	−524*	207	−2.53	0.0113
**Severe (ASA = 3)**	−335	245	−1.37	0.1718
**Very severe/fatal (ASA > 3)**	1,586*	468	3.39	0.0007
**Lenght of stay**	646*	11	58.20	< .0001
**30 days mortality**	4,113*	426	9.66	< .0001
**No. observations**	19,185			
**Model diagnostics (R**^**2**^**)**	0.63			
**Sensitivity analysis 2: All observations are included**				
**Department 1 d1**	1,084	597	1.81	0.0697
**Department 2**	883	643	1.37	0.1696
**Department 3**	−156	528	−0.30	0.7672
**Department 4**	−636	525	−1.21	0.2255
**Department 5**	−755	530	−1.43	0.1541
**Department 6**	2,096*	525	3.99	< .0001
**Department 7**	−930	871	−1.07	0.2854
**Department 8**	1,423*	541	2.63	0.0085
**Department 9**	386	544	0.71	0.4774
**Department 10**	−972	533	−1.82	0.0685
**Department 11**	6,672*	1,721	3.88	0.0001
**2006**	840*	251	3.35	0.0008
**2007**	2,036*	220	9.25	< .0001
**2008**	566*	237	2.39	0.0169
**2009**	1,324*	222	5.96	< .0001
**DRG index**	4,712*	243	19.37	< .0001
**DRG index squared**	727*	51	14.15	< .0001
**Age less than 50**	−40	267	−0.15	0.8815
**Age 50**-**60**	−43	227	−0.19	0.8513
**Age 70**-**80**	138	181	0.76	0.4458
**Age 80**-**90**	133	236	0.56	0.5733
**Age 90**+	−1,426*	650	−2.19	0.0284
**Woman**	−564*	148	−3.82	0.0001
**Daily smoker**	−106	94	−1.13	0.2583
**BMI information missing**	1,096*	450	2.44	0.0148
**Underweight**	−197	440	−0.45	0.6537
**Overweight**	−15	194	−0.08	0.9372
**Obese**	303	261	1.16	0.2445
**Very obese**	−1,350	922	−1.46	0.1433
**Diabetes**	−177	105	−1.68	0.0932
**Cerebral comorbidity**	−149	83	−1.80	0.0716
**Hypertension**	−334*	123	−2.71	0.0068
**Cardiac comorbidity**	25	53	0.46	0.6429
**Pulmonary comorbidity**	485*	176	2.75	0.0060
**Very acute admission**	2,365*	553	4.28	< .0001
**Acute admission**	1,206*	323	3.73	0.0002
**Elective**	790*	301	2.62	0.0088
**Severity information missing**	−421	407	−1.03	0.3014
**Moderate (ASA = 2)**	−480*	202	−2.37	0.0177
**Severe (ASA = 3)**	−269	238	−1.13	0.2579
**Very severe/fatal (ASA > 3)**	1,600*	460	3.48	0.0005
**Lenght of stay**	547*	10	53.62	< .0001
**30 days mortality**	3,893*	422	9.22	< .0001
**No**. **observations**	21,954			
**Model diagnostics (R**^**2**^**)**	0.62			

NOTE: Parameter estimates that are statistically significant at 5 per cent level have the suffix *.

Quality included as 30 days mortality.

In the first sensitivity analysis, parameter estimates change only marginally. This is due to few observations being excluded compared to the base case analysis. Most results remain statistically significant. In the second sensitivity analysis, based on 21,954 admissions, the overall result remains, although less parameter estimates are statistically significant. Two departments are added to this analysis, the data for these two departments were of insufficient quality for them to be included in the base case analysis above.

## Discussion

We used individual level data for identifying cost drivers at patient and department level in a sample of nine vascular surgery departments. Though the sample size at department level is small, the data set still provides a range of opportunities for analysis.

We introduce quality aspects of patient treatment into the analyses. Defining quality is a complicated task. Donabedian [28] suggests dividing the various concepts of quality into 3 major groups; structure quality, referring to the setting in which the treatment takes place, process quality, referring to the treatment as such and what is being done to the patient, and the outcome quality, referring to the effects of care. The present paper has focus on the outcome quality aspects. Due to the availability of data, the analysis is limited to a small number of quality indicators, while recognizing these measures only account for a fraction of the many outcomes entailed by hospital treatment.

For patients, we found that in particular case-mix and acute admissions were important cost drivers along with mortality and complications. At patient level, complications are associated with increased costs. Since a high level of complications express low quality, and vice versa, the complications term should be multiplied by −1 in order to comprehend the cost-quality association from the finings. If the cost-quality relation is U-shaped, our findings at patient level indicate that in this case we are on the downward sloping side of the U. This, however, is likely to be the case, since a complicated admission requires more resources. At department level, the association is not clear, but could in fact tentatively support the hypothesis of a U-shaped curve, in particular in the case of surgical complications. The sample size prevents us from concluding any further on these results.

The question of causality is, unfortunately, not resolved by the present analysis. In principle, it would be possible to include lagged department characteristics as explanatory variables in the second stage analysis. The small sample size does not, however, allow this way to establish effects that would indicate causal effects at the department level.

We estimate that the presence of wound complications is associated with an increase in patient costs by 8.4%. For comparison, estimates by the EuroDRG group show patient costs caused by wound infections in the range of 13.0% to 94.3% [13]. This seems to suggest that the production function estimated for the Danish vascular departments has characteristics comparable to the Swedish ones included in the EuroDRG study with the lowest impact of wound infections.

The findings on department level may be related to variations in hospitals reporting to the cost database. Comparison of data between hospitals is not straightforward, since the calculation of overhead costs differs between hospitals. As a result, the percentage of overhead costs included in the cost figure may vary, and department cost levels are consequently not directly comparable. The actual level of productivity may thus be affected by reporting practises. A number of other weaknesses of data should be mentioned: Data are of varying quality and the attempts we made in order to compensate for this may have led to exclusion of good data. The DRG-values we used for expressing case-mix are based on average figures and may be subject to registration errors.

Relying on a limited set of indicators of quality limits the generality of the findings. Even though mortality, complications and infections are important indicators, they are not the only ones relevant. Most importantly, the available indicators applied only cover negative aspects of health outcome, and thus leave out indicators based on positive health measures.

These weaknesses aside, the data material used in this study provides a strong basis for analysing cost drivers including salient quality variables. The data material is constructed via linkage of different data bases at patient level, made possible by the individual social security number.

We conclude that the relation between costs and quality is rather straightforward at patient level, while the department level vaguely displays an indication of a U-shaped relation between quality, in terms of surgical complications, and costs [6]. Our results suggest that rising costs for the vascular departments are associated with either increased quality or decreased quality, i.e. high quality is costly and so is low quality, nothing said about causality. Therefore, we can tentatively reject a uniformly negative association, i.e. that only low quality is costly, as suggested by [10], as such an association is only established for a minority of departments. For the relation between costs and mortality, we have not been able to identify an association at department level.

The question of whether too little or too much is spend on quality, is very complex and is not directly addressed in the literature, nor by the present analysis. However, results could be interpreted such that hospitals react to changes in quality in a rational and systematic way. Whether there is tendency over time remains to be explored.

## Competing interests

The authors declare that they have no competing interests.

## Authors’ contributions

MK drafted the first version of the paper and conducted the analyses in dialogue with JC. JC finalised the paper. Both authors read and approved the final manuscript.
